# Clinical evaluation of the efficacy of *Vitex agnus-castus* compared with cabergoline for the treatment of pseudopregnancy in the bitch

**DOI:** 10.3389/fvets.2026.1862418

**Published:** 2026-06-10

**Authors:** Viola Zappone, Romina Marcoccia, Debora Teresa Gattuso, Temy Coppola, Antonio Lombardo, Simona Di Pietro, Angela Polisca, Alessandro Troisi, Marco Quartuccio, Giulio Guido Aiudi

**Affiliations:** 1 Department of Veterinary Medicine, University of Messina, Messina, Italy; 2 Department of Bioscience, University of Camerino, Camerino, Italy; 3 Ambulatorio Veterinario NORAH, Reggio Calabria, Italy; 4 Department of Veterinary Medicine, University of Perugia, Perugia, Italy; 5 Department of Veterinary Medicine, University of Bari “Aldo Moro”, Bari, Italy

**Keywords:** bitches, cabergoline, canine pseudopregnancy, dopaminergic agents, physiotherapy, *Vitex agnus-castus*

## Abstract

Pseudopregnancy in dogs is a common condition in non-pregnant bitches. It is characterized by variable physical and behavioral signs, mainly relating to elevated prolactin levels during late dioestrus. This study aimed to evaluate and compare the clinical efficacy and tolerability of a dry extract of *Vitex agnus-castus* with cabergoline for treating pseudopregnancy. A total of 100 bitches with a clinical diagnosis of pseudopregnancy were enrolled and randomly assigned to one of two groups: *Vitex agnus-castus* group (VAC group) (*n* = 50), which was treated with 40 mg/kg orally twice daily for seven days, and cabergoline group (CAB group) (*n* = 50), which was treated with 5 μg/kg orally once daily for seven days. Subjects were assessed at T_0_, T_7_, T_14_, and T_30_ using a semi-quantitative clinical score based on physical and behavioral factors. Serum prolactin concentrations were measured at T_0_, T_7_, and T_14_. Both treatments resulted in marked and progressive reduction in clinical signs over time, with complete remission at T_30_. No clinically relevant differences were observed between the groups regarding clinical scores. In both groups, there was a significant decrease in serum prolactin levels. This decrease was particularly rapid in the CAB group, as evidenced by the results at T_7_. Adverse effects were significantly more prevalent in the CAB group than in the VAC group (32% vs. 14%). In conclusion, *Vitex agnus-castus* demonstrated comparable clinical efficacy to cabergoline and minor adverse effects, supporting its potential role as a valid therapeutic alternative in the management of canine pseudopregnancy.

## 1 Introduction

Canine pseudopregnancy, also known as pseudocyesis or false pregnancy, is a common condition in non-pregnant bitches during the late stage of oestrus or the early stage of anoestrus. It is characterized by varying degrees of mammary gland development, lactation, and maternal behavior (1).

In the domestic dog, pseudopregnancy appears to be closely related to the physiology of the oestrus cycle. Following the pre-ovulatory peak in Luteinizing Hormone (LH) (2), the luteal phase is prolonged and characterized by progesterone (P_4_) secretion from the corpus luteum. In the absence of pregnancy, luteal regression is slow and passive, resulting in the persistence of physiological oestrus (3). A progressive decrease in P_4_ is accompanied by an increase in prolactin (PRL), which causes the onset of clinical signs of pseudopregnancy (2, 4–6).

Pseudopregnancy usually appears between 6 and 20 weeks after oestrus, peaking around the 14^th^ week (2). The clinical presentation can range from latent forms that are detectable only through slight changes in mammary glands, to overt forms characterized by mammary engorgement, galactostasis, and maternal behaviors such as nesting behavior, and adopting toys, and hyper-attachment (7, 8). The signs generally resolve spontaneously within 2–4 weeks, but they may persist or leading to mastitis (7, 9–11).

Mild cases generally do not require drug treatment. Limiting mammary stimulation and discouraging maternal behaviors is sufficient (7). However, for more severe or persistent cases, targeted aetiological intervention aimed at reducing PRL levels is necessary. In this context, dopamine agonists, particularly cabergoline, are the preferred treatment due to its high efficacy and tolerability (8, 12). Other drugs, such as bromocriptine or metergoline, have also been used. However, they are associated with more gastrointestinal side effects and lower specificity (13, 14).

Despite the efficacy of dopaminergic therapy, there is growing interest in alternative approaches, including phytotherapeutic ones. Cabergoline is highly effective in reducing prolactin secretion and resolving clinical signs of pseudopregnancy; however, the availability of additional therapeutic options remains relevant because treatment choice in clinical practice may be influenced by adverse effects, patient tolerability, owner compliance, and the preference for conservative or complementary approaches. In this context, well-tolerated phytotherapeutic compounds with potential prolactin-modulating activity may represent useful alternatives or adjunctive options.

*Vitex agnus-castus L.*, also known as chaste tree, is a medicinal plant that has been extensively studied for its ability to modulate prolactin secretion and reproductive endocrine function. These effects are attributed to the presence of flavonoids, glycosylated iridoids, and labdane diterpenes with dopaminergic activity (15–20). These compounds can modulate prolactin secretion and regulate the hypothalamic–pituitary–gonadal axis, which justifies the plant's use in treating hyperprolactinaemia-related disorders (21, 22). Clinical and pharmacological studies have revealed the phytocomplex to have a favorable safety profile, characterized by mild and reversible adverse effects, and good tolerability. This suggests the potential usefulness of chaste tree as a complementary or alternative therapeutic option in the treatment of pseudopregnancy in bitches (23).

The aim of this study is to evaluate, for the first time, the clinical efficacy and tolerability of a standardized dry extract of *Vitex agnus-castus* in the treatment of pseudopregnancy in bitches. The study will compare its effects with those of cabergoline, which is currently considered the standard treatment for this condition.

## 2 Materials and methods

### 2.1 Ethical approval

All treatments, housing conditions and animal care were carried out in accordance with EU Directive 2010/63/EU on the protection of animals used for scientific purposes. The Ethics Committee of the Department of Veterinary Medicine and Animal Production at the University of <city>Messina</city>, Italy, approved the protocol and procedures (protocol no. 19/2026). Informed consent was obtained from each dog owner prior to inclusion in the study.

### 2.2 Animals and study design

A total of 100 client-owned unspayed bitches of various breeds, with an average age of 4.52 years ± 1.86 years, were included in the study, which took place from June 2022 to January 2026. All dogs were housed indoors, kept in rooms with adequate natural light exposure, and fed a commercial diet. The bitches were presented for a clinical examination with signs consistent with pseudopregnancy.

At the time of enrolment (T_0_), all dogs underwent a clinical assessment comprising a general and reproductive history, a general physical examination, and an assessment of the mammary glands. For each subject, anamnestic and clinical data were recorded including age, breed, body weight, reproductive status, time elapsed since the last oestrus, any previous episodes of pseudopregnancy, and the duration of clinical signs prior to enrolment.

Bitches exhibiting active lactation and/or mammary hyperplasia, accompanied by at least one behavioral sign indicative of pseudopregnancy (e.g., nest-building, adopting toys or restlessness), were deemed eligible. Those with confirmed pregnancy, lactation following a recent birth, mastitis, mammary neoplasms or significant systemic diseases were excluded. Bitches that had received treatment with dopaminergic agonists or other drugs capable of interfering with the study design within the previous 30 days were also excluded.

**Figure 1:**
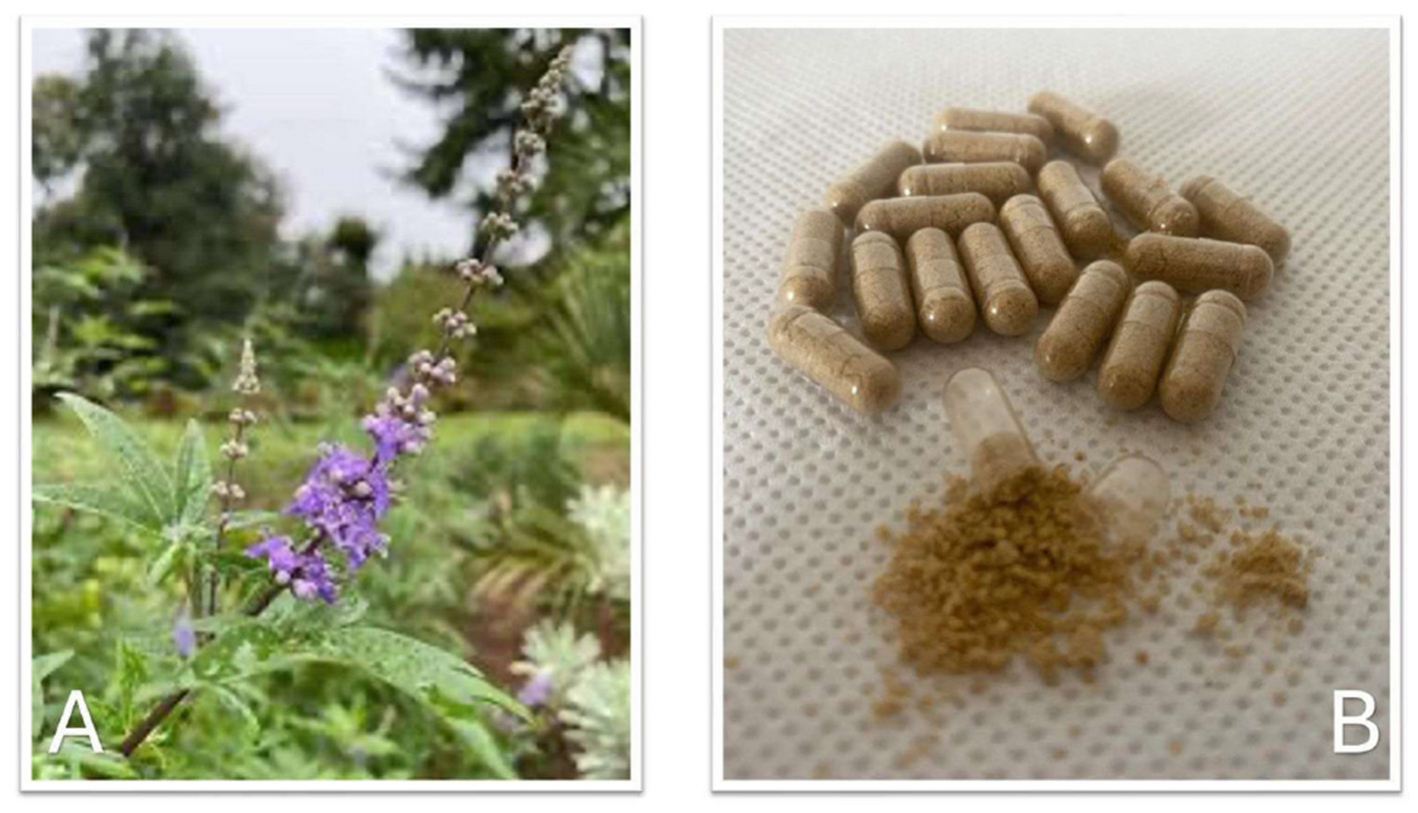
*Vitex agnus-castus* plant **(A)**. *Vitex agnus-castus* dry extract used in this study **(B)**.

The subjects were randomly assigned to one of two treatment groups: the *Vitex agnus-castus* group (*n* = 50) and the cabergoline group (*n* = 50). The VAC group received a galenic preparation in capsules containing a dry extract of *Vitex agnus-castus*, standardized and titrated to 0.6% agnuside and characterized by a 4:1 drug-to-extract ratio, along with a thickening agent (1,5 gr of hydroxypropyl methylcellulose) and 2.5 mg of flaxseed meal (Figure 1). The extract, obtained through hydroalcoholic extraction with 60% v/v ethanol in accordance with the European Pharmacopeia, was administered orally at a dose of 40 mg/kg twice daily for 7 days. The CAB Group received cabergoline (Galastop^®^, Ceva Salute Animale S.p.A., Milano, Italy) orally at a dose of 5 μg/kg once daily for 7 days. Owners received standardized instructions regarding treatment administration and home care, focusing on avoiding stimulation of the mammary glands. Each owner was also provided with a standardized questionnaire to be completed to record changes in feeding habits, maternal signs, and any side effects observed. Changes in feeding habits were assessed using a 4-point scale (0–3): 0 indicated no change from the usual feeding behavior; 1, a mild change; 2, a moderate change; and 3, a marked alteration in feeding habits. For maternal signs, each behavioral parameter was scored on a 4-point scale ranging from 0 to 3, where 0 indicated absence of the sign, 1 mild presence, 2 moderate presence, and 3 severe presence. The expected side effects included nausea, sialorrhoea, vomiting, diarrhea, constipation, changes in fecal consistency, pruritus, tremors, lethargy, and euphoria. Owners were instructed to report any adverse effects daily, specifying their type, time of onset, and duration. Side effects were then coded as a dichotomous variable (absent/present) for each subject.

Clinical follow-up evaluations were carried out on days 7 (T_7_), 14 (T_14_), and 30 (T_30_). At each time point, the clinical examination and assessment of the mammary gland were repeated, as were the recording of physical and behavioral scores.

Blood samples were also taken at T_0_, T_7_ and T_14_ to determine serum prolactin concentrations. This was done alongside the clinical assessment when the owners brought the animals in for a follow-up appointment.

### 2.3 Clinical assessment and scoring system

At each time point (T_0_, T_7_, T_14_, and T_30_) all dogs underwent a full clinical examination. The severity of pseudopregnancy was quantified using a standardized, semi-quantitative clinical scoring system that was developed for this study.

For each physical and behavioral parameter, a score from 0 to 3 was assigned: 0 indicated an absence of the sign; 1, a mild presence; 2, a moderate presence; and 3, a severe presence.

The physical parameters assessed included mammary gland development, milk secretion, and changes in appetite. Mammary development was assessed based on an increase in gland volume, while lactation was classified as absent (0), present only during milk expression (1), intermittent (2) or continuous (3). Changes in appetite were classified in relation to normal feeding behavior.

The behavioral parameters included the presence of nesting behavior and maternal behavior toward objects, such as adopting toys or other items, as well as restlessness and anxiety. These parameters were assessed based on clinical observation and information provided by the owners through targeted history collection.

For each subject, physical and behavioral scores were derived by summing the respective parameters. The total clinical score (CS) was subsequently calculated as the sum of the physical and behavioral scores.

### 2.4 Serum prolactin assay

Blood samples were collected from the jugular vein at T_0_, T_7_, and T_14_, always at the same time in the morning, to measure serum prolactin levels. The samples were transported to the laboratory at room temperature within one to 2 hours of collection. Serum was obtained by centrifugation at 3,000 rpm for 10 minutes, after which it was stored at −20 °C until analysis.

Serum prolactin concentrations were determined using a commercially available enzyme-linked immunosorbent assay (ELISA) kit specific for canine prolactin (MyBioSource Dog Prolactin ELISA Kit; Catalog number: MBS1609051), following the manufacturer's instructions.

### 2.5 Statistical analysis

Statistical analysis was performed using Jamovi software (version 2.7.24.0 for Microsoft) (24). The collected data were analyzed using repeated measures analysis of variance (ANOVA) to assess trends in clinical outcomes over time and the potential impact of treatment. The model included time (T_0_, T_7_, T_14_, and T_30_) as a within-subject factor and treatment group (*Vitex agnus-castus* vs. cabergoline) as a between-subject factor. For each variable of interest, represented by the physical score, behavioral score, and total clinical score, the main effects of time and group, and the time × group interaction, were examined.

Where the overall analysis revealed significant differences, *post hoc* comparisons were carried out between the different time points. Corrections for multiple comparisons were applied using Tukey's and Bonferroni's tests to control the risk of type I error. The level of statistical significance was set at *p* < 0.05 for all analyses. Effect sizes were expressed as eta-squared (η^2^), to quantify the magnitude of the observed differences and facilitate clinical interpretation.

The presence of side effects was assessed as an absence/presence dichotomous variable in the two treatment groups (*VAC* and CAB). Differences in the frequency of side effects between the groups were analyzed using the chi-squared test (χ^2^). The level of statistical significance was set at *p* < 0.05.

## 3 Results

### 3.1 Demographic results

A total of 100 nulliparous, unspayed bitches of different breeds were included in the study. The mean age was 4.38 ± 1.77 years in the *VAC* group and 4.67 ± 1.95 years in the CAB group. Mean body weight was 14.92 ± 3.78 kg and 16.14 ± 3.51 kg, respectively.

From an anamnestic perspective, 62% of the bitches had a history of pseudopregnancy. In 38% of cases, this was the first clinical manifestation, indicating a significant prevalence of recurrent cases within the cohort (Table 1).

**Table 1:** Demographic and clinical characteristics of the bitches included in the study across the two treatment groups (*VAC* and CAB).

Groups	*Vitex agnus-castus*	Cabergoline
Number of subjects	50	50
Age (years)	4.38 ± 1.77	4.67 ± 1.95
Weight (Kg)	14.92 ± 3.78	16.14 ± 3.51
Previous episodes of pseudopregnancy, *n* (%)	39 (78.0%)	24 (48.0%)
Baseline physical score (T_0_)	6.10 ± 1.52	6.18 ± 1.41
Baseline behavioral score (T_0_)	7.90 ± 0.84	7.34 ± 1.14
Baseline total clinical score (T_0_)	14.00 ± 1.77	13.52 ± 1.59

Data are presented as mean ± standard deviation or as a number (percentage).

### 3.2 Physical score

**Figure 2:**
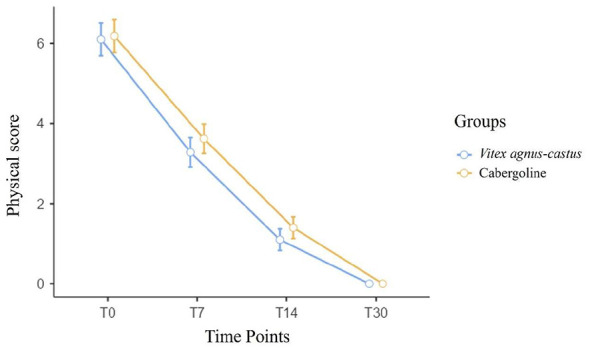
Physical score trends in the two treatment groups (*VAC* and CAB) at different time points (T_0_, T_7_, T_14_, T_30_). Data are expressed as mean ± standard error.

Descriptively, mean physical scores (expressed as mean ± standard error) were comparable between the two groups at baseline T_0_ (*VAC*: 6.10 ± 0.21; CAB: 6.18 ± 0.19), indicating similar initial symptom severity. Over the follow-up period, both groups exhibited a progressive reduction in physical scores, with improvement already evident at T_7_ (*VAC*: 3.28 ± 0.20; CAB: 3.62 ± 0.15) and becoming more pronounced at T_14_ (*VAC*: 1.10 ± 0.15; CAB: 1.40 ± 0.11). By T_30_, clinical signs had completely resolved in both groups (Figure 2).

Analysis of the physical score revealed a significant and progressive reduction in clinical signs over time. Repeated measures ANOVA showed a highly significant effect of time, indicating that the severity of the physical signs of pseudopregnancy decreased substantially throughout the observation period. The associated effect size was very high (η^2^ = 0.821), suggesting that time was the main determinant of clinical improvement. The reduction in physical score occurred at a comparable rate in both groups, with no significant difference between bitches treated with *Vitex agnus-castus* and those treated with cabergoline.

*Post-hoc* comparisons confirmed that the reduction in the physical score was significant at all considered time points. Improvement was evident at T_7_ compared with baseline (T_0_), continuing significantly at T_14_ and T_30_. Comparisons between the intermediate time points were also all statistically significant, indicating a progressive and continuous trend in the remission of physical symptoms. This pattern was consistent across both treatment groups.

### 3.3 Behavioral score

**Figure 3:**
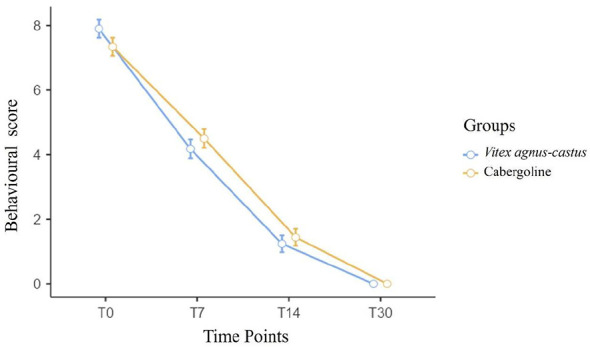
Behavioral score trends in the two treatment groups (*VAC* and CAB) at different time points (T_0_, T_7_, T_14_, T_30_). Data are expressed as mean ± standard error.

Mean baseline values (expressed as mean ± standard error) were comparable between the two groups (*VAC*: 7.90 ± 0.12; CAB: 7.34 ± 0.16), indicating similar behavioral involvement in pseudopregnancy at the outset (T_0_). Over time, both groups exhibited a rapid and marked reduction in behavioral scores, with improvement already evident at T_7_ (*VAC*: 4.18 ± 0.15; CAB: 4.50 ± 0.14) and becoming more pronounced at T_14_ (*VAC*: 1.24 ± 0.12; CAB: 1.44 ± 0.14). Complete resolution was observed in both groups by T_30_ (Figure 3).

Regarding the behavioral score, the results showed an even more pronounced trend than that observed for the physical parameters. The repeated-measures ANOVA revealed a highly significant time effect, with an extremely large effect size (η^2^ = 0.920), indicating a drastic reduction in behavioral symptoms during the follow-up period.

As with the physical score, no significant main effect of the treatment group was observed. However, a statistically significant time × group interaction was detected. Nevertheless, the associated effect size was very small (η^2^ = 0.003), suggesting that, while this difference is statistically detectable, it is not clinically relevant.

*Post hoc* comparisons revealed a significant reduction in the behavioral score at all time points. A clear improvement was evident by T_7_, with further improvement observed at T_14_ and T_30_. No significant differences were observed between the two groups at individual assessment points, confirming that both treatments effectively reduce the behavioral symptoms associated with pseudopregnancy.

### 3.4 Total clinical score

**Figure 4:**
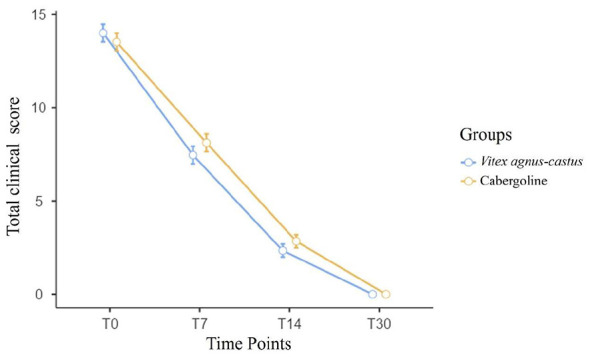
Total score trends in the two treatment groups (*VAC* and CAB) at various time points (T_0_, T_7_, T_14_, T_30_). Data are expressed as mean ± standard error.

The total clinical score (expressed as mean ± standard error), which combines physical and behavioral parameters, was similar for both groups at the start of the study (*VAC*: 14.00 ± 0.25; CAB: 13.52 ± 0.22). During the follow-up period, both treatments resulted in substantial reduction in the total score. Improvement was already evident at T_7_ (*VAC*: 7.46 ± 0.27; CAB: 8.12 ± 0.19) and was further accentuated at T_14_ (*VAC*: 2.34 ± 0.18; CAB: 2.84 ± 0.16). Complete clinical remission was achieved by T_30_ (Table 2; Figure 4).

**Table 2:** Trend in clinical scores (physical, behavioral and total clinical) in the two treatment groups (*VAC* and CAB) at different time points (T_0_, T_7_, T_14_, T_30_).

Outcome	Time	*Vitex agnus-castus*	Cabergoline
Physical score	T_0_	6.10 ± 0.21	6.18 ± 0.19
	T_7_	3.28 ± 0.20	3.62 ± 0.15
	T_14_	1.10 ± 0.15	1.40 ± 0.11
	T_30_	0.00 ± 0.00	0.00 ± 0.00
Behavioral score	T_0_	7.90 ± 0.12	7.34 ± 0.16
	T_7_	4.18 ± 0.15	4.50 ± 0.14
	T_14_	1.24 ± 0.12	1.44 ± 0.14
	T_30_	0.00 ± 0.00	0.00 ± 0.00
Total clinical score	T_0_	14.00 ± 0.25	13.52 ± 0.22
	T_7_	7.46 ± 0.27	8.12 ± 0.19
	T_14_	2.34 ± 0.18	2.84 ± 0.16
	T_30_	0.00 ± 0.00	0.00 ± 0.00

Data are expressed as mean ± standard error.

Repeated measures ANOVA of the total clinical score confirmed and reinforced the findings observed in previous analyses. A highly significant effect of time was indeed observed, with a very high effect size (η^2^ = 0.938), indicating a marked overall reduction in symptoms throughout the treatment period.

No significant effect of the treatment group was detected, while the time × group interaction was statistically significant. However, the effect size associated with this interaction was extremely small (η^2^ = 0.002), indicating a negligible clinical difference.

*Post hoc* comparisons revealed a significant reduction in the total score at all analyzed time points. Improvement was evident at T_7_ compared with baseline, with further significant reductions at T_14_ and T_30_. The pattern of improvement was continuous and progressive over time, with no significant differences observed between the two groups at any individual assessment time point.

### 3.5 Serum prolactin

Analysis of serum prolactin concentrations revealed a significant and progressive decrease in levels over time in both treatment groups. At baseline (T_0_), mean prolactin levels were similar in bitches treated with *Vitex agnus-castus* (38.78 ± 4.24 ng/ml) and in those treated with cabergoline (38.38 ± 4.56 ng/ml).

**Figure 5:**
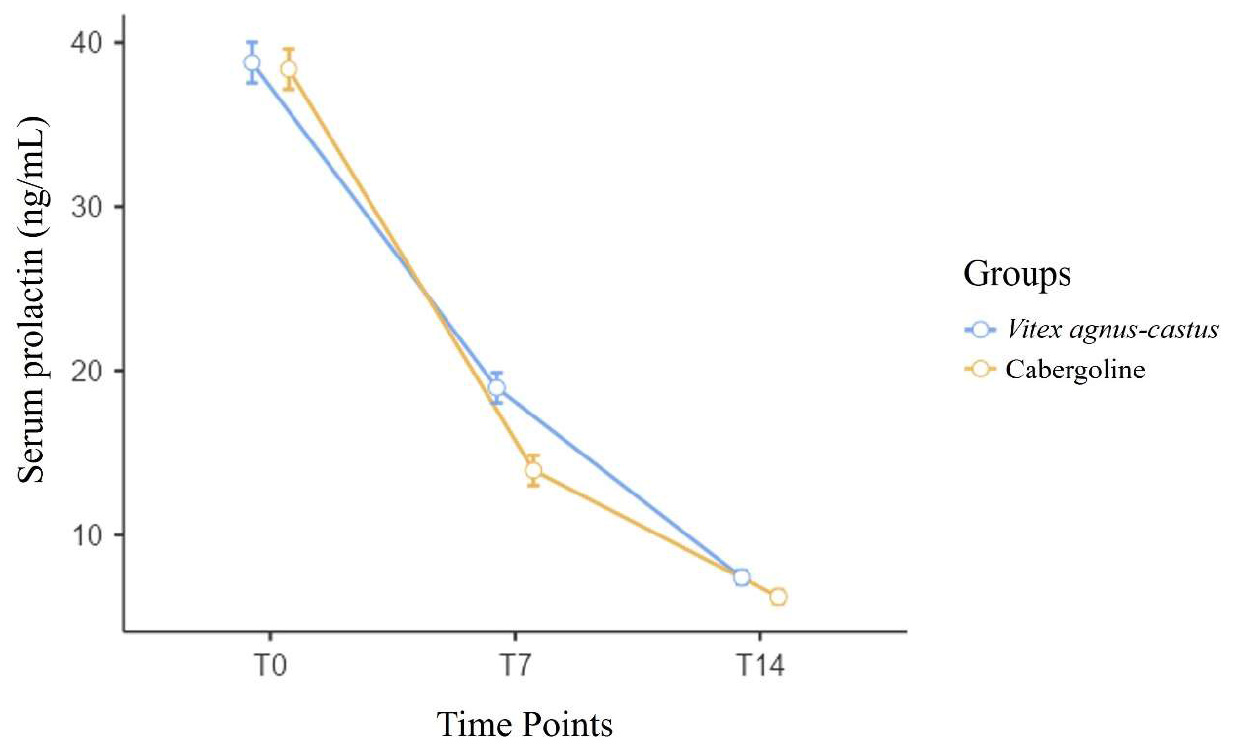
Serum prolactin concentrations in the two treatment groups (*VAC* and CAB) at various time points (T_0_, T_7_, T_14_). Data are expressed as mean ± standard deviation.

During the follow-up period, a significant reduction in prolactin concentrations was observed from time point T_7_ onwards. The decrease was more pronounced in the CAB group (13.92 ± 3.68 ng/ml) than in the *VAC* group (18.96 ± 2.79 ng/ml). Although this difference was initially statistically significant, it gradually diminished over time. By T_14_, prolactin levels had further decreased in both groups (*VAC*: 7.40 ± 1.43 ng/ml; Cabergoline: 6.22 ± 1.51 ng/ml), with a smaller difference between the two groups (Figure 5).

Analysis using repeated-measures ANOVA revealed a highly significant effect of time (*p* < 0.001, η^2^ = 0.933), indicating a dramatic reduction in prolactin concentrations within the observation period. A significant treatment group effect (*p* < 0.001) was also observed, as well as a statistically significant time × group interaction (*p* < 0.001). However, the effect sizes associated with the group factor (η^2^ = 0.006) and the interaction (η^2^ = 0.005) were very small.

### 3.6 Side effects

Analysis of the frequency of side effects revealed a difference between the two treatment groups. Specifically, 14% (7 out of 50) of subjects in the *VAC* group reported side effects, compared to 32% (16 out of 50) in the CAB group (Table 3).

**Table 3:** Frequency of side effects in the two treatment groups (*VAC* and CAB).

Group	Total *n*	Adverse effects, *n* (%)	No side effects, *n* (%)
*Vitex agnus-castus*	50	7 (14.0%)	43 (86.0%)
Cabergoline	50	16 (32.0%)	34 (68.0%)

The data are expressed as a number (percentage).

The side effects recorded in the CAB group were mainly nausea and/or vomiting, which occurred in the first few days of treatment. In the *VAC* group, loose stools were observed, also appearing in the first few days of treatment.

The chi-squared test revealed a statistically significant difference between the two groups [χ^2^(1) = 4.57, *p* = 0.032].

## 4 Discussion

To the best of our knowledge, this is the first study comparing *Vitex agnus-castus* and cabergoline for the treatment of canine pseudopregnancy. The findings indicate that both treatments significantly reduce clinical signs over time, with no statistically significant differences between groups. These results suggest that the phytotherapeutic approach is clinically comparable to standard pharmacological treatment and provide further evidence supporting the use of *Vitex agnus-castus* in veterinary practice.

*Vitex agnus-castus* is a plant with a complex composition including flavonoids, iridoid glycosides, diterpenoids, phenolic compounds, and bioactive fatty acids (16, 17, 20, 25). The most representative constituents include casticin, apigenin, vitexin, isovitexin, luteolin, orientin, penduletin and the iridoid glycosides agnuside and aucubin (26, 27). The presence of linoleic acid, which has been identified as a selective ligand for estrogen receptors, helps to explain the plant's endocrine-modulating activity. This activity includes oestrogenic, progestogenic and dopaminergic effects, resulting in reduced prolactin levels and modulation of luteal hormones (21, 28). Meanwhile, the extract's high antioxidant and anti-inflammatory capacity, conferred by its high content of flavonoids and phenolic compounds, has been extensively demonstrated in experimental models (29–31). Several diterpenoids and lignans exhibit cytotoxic, anticancer and immunomodulatory activities, which contribute to the pharmacological versatility of the phytocomplex (32–34). There are also antimicrobial and antifungal properties (35–37), as well as antinociceptive and opioidergic properties (26, 38). Other properties include antiepileptic properties (39) and osteoprotective and bone-healing properties (40, 41). Furthermore, there are hepatoprotective effects in the prevention of non-alcoholic fatty liver disease (42).

Canine pseudopregnancy is a condition closely related to the physiology of the oestrus cycle, particularly the endocrine dynamics of the luteal phase. In non-pregnant bitches, the absence of an endogenous luteolytic mechanism results in the persistence of the corpus luteum and prolonged progesterone secretion. As luteal function gradually regresses during mid-to-late diestrus, circulating progesterone concentrations progressively decrease. This endocrine shift is accompanied by an increase in prolactin secretion, which contributes to the development of mammary enlargement, lactation, and maternal behaviors characteristic of pseudopregnancy (2, 3). Prolactin is responsible for mammary development, lactation, and maternal behaviors, and is under the inhibitory control of hypothalamic dopamine. However, the relationship between circulating prolactin levels and clinical manifestations is not always linear, likely due to individual differences in tissue sensitivity or the presence of different molecular isoforms of the hormone, each with distinct biological activity (43). This helps to explain the wide clinical variability observed among affected individuals.

Pseudopregnancy is a common, yet probably underdiagnosed, condition in veterinary practice. Several studies report that a significant proportion of unspayed female dogs may exhibit clinical signs consistent with this condition, with marked variability in the types and severities of manifestations (44). The most common physical signs are mammary gland development and lactation, while behavioral signs include nest-building, adopting toys, and displays of maternal behavior. These behaviors are sometimes associated with aggression or restlessness (7, 8). It is important to emphasize that behavioral signs may often be predominant, or even the only manifestation, making diagnosis less straightforward and potentially leading to underestimation (43). This clinical heterogeneity, combined with the often self-limiting nature of pseudopregnancy, contributes to variability in therapeutic approaches and failure to identify the condition in routine clinical settings. Consequently, the actual incidence of pseudopregnancy may be higher than reported in literature.

Identifying an effective phytotherapeutic alternative to cabergoline is particularly relevant, especially in light of the limitations associated with dopaminergic treatments. *Vitex agnus-castus* is characterized by a favorable safety profile, with generally mild, transient, and reversible adverse effects, as well as a low potential for drug interactions (23). Recent pharmacological evidence further supports its use, as experimental studies in both rats and humans have shown that specific diterpenes and triterpenes present in the extract can activate D_2_ dopaminergic receptors, thereby exerting dopamine-like activity and contributing to reduced prolactin secretion (22, 45, 46). In particular, compounds such as viteagnusin I and certain pentacyclic triterpenes have demonstrated significant agonist activity at these receptors, providing a plausible molecular mechanism for the observed clinical effects (22, 46). These characteristics make *Vitex agnus-castus* a promising therapeutic option, particularly in cases where treatment tolerability, owner compliance, or the preference for more conservative approaches play a key role in clinical decision-making.

The endocrine findings of the present study further support this pathophysiological rationale. In both treatment groups, serum prolactin concentrations showed a marked and progressive reduction over time, with a highly significant effect of time and a very large effect size. These results confirm that both cabergoline and *Vitex agnus-castus* effectively modulate the dopaminergic–prolactin axis, thereby contributing to the resolution of clinical signs.

Although a more rapid reduction in prolactin levels was observed in the cabergoline-treated group at T_7_, this difference progressively diminished during the follow-up period and was of limited magnitude at later time points. Moreover, the effect sizes associated with both the group factor and the time × group interaction were extremely small, suggesting that, despite statistical significance, these differences are unlikely to be clinically meaningful.

These results are consistent with previous reports. In a study by Mogheiseh et al., which compared the effects of *Vitex agnus-castus* and cabergoline on oestrus induction in bitches, no significant differences in prolactin concentrations were observed between treatment groups (47). Similarly, in the present study, no meaningful differences were detected between *Vitex agnus-castus* and cabergoline in the modulation of prolactin levels, despite statistically significant effects for the group factor and the time × group interaction, both associated with extremely small effect sizes.

Both treatments, *Vitex agnus-castus* and cabergoline, were associated with a significant reduction in clinical signs over time, with no statistically significant differences between groups. Time emerged as the main determinant of clinical improvement, with very large effect sizes, indicating a progressive remission of symptoms. While this pattern is consistent with the self-limiting nature of pseudopregnancy, it also suggests that both treatments may contribute to accelerating symptom resolution.

The efficacy of cabergoline observed in this study is consistent with its established role as a first-line treatment for pseudopregnancy, owing to its action as a direct dopamine agonist and the consequent inhibition of prolactin secretion (1, 4, 12, 48). Regarding *Vitex agnus-castus*, the present findings are supported by pharmacological evidence suggesting dopaminergic activity. While this effect has traditionally been considered indirect, recent studies indicate that it may be at least partly mediated by a direct interaction between phytochemicals and D_2_ receptors, with downstream modulation of intracellular pathways such as adenylate cyclase inhibition and reduced cAMP production (46).

This evidence supports a reinterpretation of the mechanism of action of chaste tree, positioning it at the interface between phytotherapy and classical receptor pharmacology. Its main bioactive compounds, including flavonoids and diterpenes, have been shown to interact with D_2_ dopaminergic receptors, leading to reduced prolactin secretion (22, 23). This mechanism is consistent with findings from human clinical studies, in which *Vitex* extracts have demonstrated efficacy in lowering prolactin levels and improving conditions associated with hyperprolactinaemia, in some cases showing effects comparable to dopaminergic drugs (49). Previous evidence further supports the role of *Vitex agnus-castus* in the modulation of reproductive endocrine function and prolactin secretion. Recent reviews and pharmacological studies have summarized evidence indicating that *Vitex agnus-castus* extracts may reduce prolactin secretion through dopaminergic mechanisms, mainly involving interaction with dopamine D2 receptors (22, 46, 49). In human medicine, this prolactin-lowering activity has been investigated in patients with hyperprolactinemia and prolactin-related reproductive disorders. In a randomized controlled trial comparing *Vitex agnus-castus* with bromocriptine in women with hyperprolactinemia, both treatments significantly reduced prolactin concentrations, with no significant difference between groups after treatment; however, adverse effects were more frequent in the bromocriptine group. Although these findings cannot be directly extrapolated to canine pseudopregnancy, they support the prolactin-modulating potential of *Vitex agnus-castus* and strengthen the rationale for its evaluation in prolactin-dependent reproductive conditions (50).

Experimental studies suggest that *Vitex agnus-castus* may modulate the hypothalamic–pituitary–gonadal axis, influencing LH, follicle-stimulating hormone (FSH), estrogen, progesterone, and prolactin secretion (51). Notably, its endocrine effects appear to be dose-dependent: lower doses have been associated with decreased estrogen levels and increased progesterone and prolactin concentrations, possibly through inhibition of FSH release and stimulation of LH secretion, whereas higher doses seem to reduce prolactin levels without significantly affecting LH and FSH (22, 52–56). This dose-dependent relationship may help explain the apparently paradoxical effects of *Vitex agnus-castus*, whereby low doses may stimulate milk production, while higher doses exert the opposite effect by reducing prolactin secretion. Such variability may contribute to differences in clinical outcomes and should be carefully considered in veterinary practice.

The absence of significant differences between the two treatments suggests that *Vitex agnus-castus* and cabergoline are clinically comparable. This finding is in line with the study by Mogheiseh et al., which reported similar endocrine and clinical effects of *Vitex agnus-castus* and cabergoline in dogs (47). Although the mechanism of action of chaste tree is likely less direct than that of cabergoline, available evidence indicates that modulation of prolactin, even through indirect pathways, is sufficient to achieve a meaningful clinical improvement.

From a clinical perspective, these results suggest that *Vitex agnus-castus* may represent a valid therapeutic option in the management of canine pseudopregnancy. Its use may be particularly appropriate in cases where cabergoline is poorly tolerated or when a phytotherapeutic approach is preferred. Moreover, the favorable safety profile of chaste tree, characterized by generally mild and reversible adverse effects and a low potential for drug interactions, represents an additional advantage (23). In our study cabergoline was associated with a higher incidence of adverse effects compared with *Vitex agnus-castus*, primarily consisting of nausea and vomiting during the first few days of treatment. In contrast, adverse effects observed in the *Vitex agnus-castus* group were less frequent and less severe, mainly involving transient episodes of loose stools. These findings are consistent with previous reports indicating that dopamine agonists are commonly associated with gastrointestinal side effects, whereas *Vitex agnus-castus* is generally well tolerated (13, 23). This difference in safety profile may represent a key factor in treatment selection, particularly in more sensitive individuals or in cases where tolerability is a primary concern.

The present study has several limitations that should be considered when interpreting the results. The assessment of behavioral parameters relied, at least in part, on owner-reported observations, introducing a potential element of subjectivity. An additional limitation is the use of a semiquantitative clinical scoring system specifically developed for this study and not previously validated, which may affect the reproducibility and generalizability of the findings. Finally, although the duration of follow-up was adequate for assessing short-term clinical response, it does not allow conclusions to be drawn regarding the potential occurrence of long-term recurrences.

## 5 Conclusion

The results of the present study indicate that both cabergoline and *Vitex agnus-castus* are associated with a significant reduction in clinical signs of canine pseudopregnancy over time, with a comparable pattern of improvement between the two treatments. Analysis of serum prolactin concentrations confirmed that both interventions lead to a marked decrease in hormone levels, supporting their effect on modulation of the dopaminergic–prolactin axis. In both groups, complete clinical remission was achieved within 30 days, confirming the efficacy of both therapeutic options in the short-term management of the condition. Within this context, *Vitex agnus-castus* demonstrated a more favorable tolerability profile compared with cabergoline, with a lower frequency of side effects and a reduced incidence of gastrointestinal adverse events. This finding, together with the observed clinical efficacy, suggests that the phytocomplex may represent a valid therapeutic alternative to cabergoline. *Vitex agnus-castus* may be considered not only as an alternative option in cases of intolerance to cabergoline, but also as a potential first-line choice in selected contexts, such as mild to moderate forms of pseudopregnancy or situations in which a more conservative therapeutic approach is preferred.

## Data Availability

The original contributions presented in the study are included in the article/supplementary material, further inquiries can be directed to the corresponding author.
